# Pharmacovigilance in the treatment of cutaneous leishmaniasis: adverse event profile at a reference center

**DOI:** 10.1590/0037-8682-0038-2026

**Published:** 2026-09-18

**Authors:** Ana Luiza Silva Rezende, Janaína de Pina Carvalho, Beatriz Prado Noronha, Sarah Nascimento Silva

**Affiliations:** 1 Fundação Oswaldo Cruz, Instituto René Rachou, Núcleo de Avaliação de Tecnologias em Saúde, Pesquisa Clínica e Políticas Públicas em Doenças Infecto-Parasitárias, Belo Horizonte, MG, Brasil.

**Keywords:** Leishmaniasis, Pharmacovigilance, Quality of life, Adverse event, Adverse drug reaction

## Abstract

**Background:**

Cutaneous leishmaniasis treatment is frequently associated with adverse events (AEs). We investigated the profile of AEs and examined their associations.

**Methods:**

This prospective observational study was conducted at a Brazilian reference center to describe AEs according to their occurrence rate, severity, and seriousness.

**Results:**

Among 318 AEs recorded in 92 patients, the highest AE occurrence rate was among those treated with miltefosine. The highest rate of serious AEs was among patients treated with liposomal amphotericin B. Local therapy using meglumine antimoniate showed a more favorable safety profile.

**Conclusion:**

The different profiles highlight the importance of pharmacovigilance in real-world settings.

Cutaneous leishmaniasis (CL) is a neglected noncontagious infectious disease most prevalent in tropical countries^1^ ^,^ ^2^. Treatment options for CL are limited, show variable efficacy, and are associated with significant toxicity, making treatment choice dependent on safety considerations and requiring medical specialists follow-up during treatment^1^.

Pentavalent antimonials, amphotericin B formulations, and miltefosine are among the most used drugs to treat CL, each presenting with distinct adverse event (AE) profiles. Cardiotoxicity and laboratory abnormalities are well described for systemic antimonials, while the use of amphotericin B is associated with infusion-related reactions and organ toxicity, and the use of miltefosine frequently causes gastrointestinal intolerance and carries teratogenic risk. Pharmacovigilance therefore plays a key role in monitoring CL treatment: Characterizing the safety profile enables timely and appropriate management and thus contributes to improved therapeutic effectiveness and patient safety^1^ ^,^ ^3^. However, the evidence for AEs related to CL treatment is scarce and methodologically heterogeneous, limiting comparability across studies.

This prospective observational study aimed to describe the AE profile observed at a CL reference center, and to evaluate its association with sociodemographic, lifestyle, clinical, and quality of life variables.

The study was conducted between October 2020 and April 2023 at a leishmaniasis reference center in Minas Gerais, a state in Brazil that is endemic for CL. The center provides specialized and free care through the Unified Health System. Using convenience sampling, we enrolled 57.3% of the patients who tested positive for leishmaniasis and whose cases were reported by the service during this period.

Eligible patients were aged older than 12 years with confirmed CL, were undergoing treatment at the center, and provided consent to participate. Exclusion criteria were not predefined. According to routine procedures, participants were followed and monitored throughout their treatment until the assessment period for cure, which was defined as 180 days after the start of treatment. 

Sociodemographic, lifestyle, and clinical data were obtained from patients’ physical and electronic medical records. Quality of life data were extracted from a concomitant study conducted in the same population; in the concomitant study, the EuroQol EQ-5D-3L instrument was administered to patients during treatment or within 30 days of treatment initiation^4^. This study was approved by the institution’s ethics committee (CAAE 28929220.0.0000.5091) and this manuscript was prepared in accordance with the STROBE guidelines (Supplementary Table S1).

AEs were the main outcome and were described according to the terminology of the Medical Dictionary for Regulatory Activities version 28.0^5^ and classified by System Organ Classes. For severe cases, AEs were also classified by the High-Level Group Term. Severity was graded according to The Division of AIDS (DAIDS) Table for Grading the Severity of Adult and Pediatric Adverse Events, version 2.1^6^, whereas seriousness was assessed according to the classification of the Brazilian Health Regulatory Agency^7^. AEs with a severity grade of ≥3 were also considered serious because hospitalization is recommended from this level upward^6^ ^,^ ^7^. Causality in relation to treatment was assessed using the World Health Organization-Uppsala Monitoring Center (WHO-UMC) algorithm^8^. AEs classified as certain, probable, or possible were considered treatment-related, whereas those classified as unlikely, conditional or unclassified, and unclassifiable were considered nontreatment-related. For patients who underwent more than one therapeutic cycle, only the cycle with the highest number of AEs was considered; for patients with the same number of events, the most recent cycle was used.

Exploratory variables included sociodemographic characteristics, lifestyle factors, clinical variables, and quality of life measures. Sociodemographic information comprised sex, age group, and educational level. Lifestyle factors included alcohol consumption. Clinical variables included type of case (new), clinical form of the disease, presence of comorbidities, polypharmacy, number of lesions, treatment administered, and treatment discontinuation. Potential drug-drug interactions among the medications used by patients were verified using the Micromedex® platform (version 4.6.5; 2024; Merative, Ann Arbor, MI, USA).

A descriptive analysis was conducted for the sociodemographic, lifestyle, clinical, and quality of life characteristics of eligible participants who started treatment at the reference center. AEs were described according to their inherent classifications (System Organ Classes, High-Level Group Term, severity, seriousness, and causality) and the treatment regimen. Categorical variables are reported as absolute and relative frequencies, and continuous variables are summarized using measures of central tendency and dispersion. AEs are presented as absolute counts and occurrence rates (number of events divided by the number of treated patients). AE rates were also stratified by treatment regimens, considering only those regimens used by more than 10 patients.

A logistic regression was proposed to complement the description of the profile found in this population. Based on the size of the subgroups and the risk of indication biases, this analysis was limited to the main variables of the study, without the possibility of stratifying some conditions. The AE variable was converted into a binary outcome based on event occurrence, with the group with no AEs defined as the reference category. The adjusted model included sociodemographic, lifestyle, and clinical variables. For the clinical variable “number of lesions,” the analysis included only patients with the cutaneous form of the disease; patients with mucocutaneous lesions were grouped with those in the mucosal category^1^. All analyses were performed using IBM SPSS Statistics for Windows (version 30.0; IBM Corp., Armonk, NY, USA). A significance level of 5% (p ≤ 0.05) was adopted for all statistical tests.

Of 152 patients initially invited to participate in the study, 143 were enrolled. Among these, one patient was clinically cured before starting treatment, and three did not return to the reference center, although they had received a prescription; these individuals were therefore excluded from subsequent analyses. The final sample thus comprised 139 patients, with no loss to follow-up. Some variables had missing responses, which led to variation in the total number of observations across the characteristics analyzed.

The profile of patients treated at the center showed a predominance of male participants (74.1%), with a mean age of 51 years (standard deviation = 17.3). The cutaneous form of CL (81.3%) predominated. In total, 318 AEs were recorded in 92 patients, corresponding to an incidence of 66.2% in the analyzed sample, with a mean of 2.0 (SD = 2.3) events per patient. Most reported AEs were classified as nonserious (94.7%) and related to the treatment (certain, probable, or possible; 95.3%; Table 1). Gastrointestinal disorders were the most frequent System Organ Classes, accounting for 25.1% of AEs, and were predominant among those treated with miltefosine (76.2%; Table 2).

**TABLE 1:** Sociodemographic characteristics, lifestyle, and clinical features of the sample (N = 139 patients).

Variable	Number	%
**Sex**		
Female	36	25.9
Male	103	74.1
**Age group (years)**		
15 to 29 years	20	14.4
30 to 59 years	66	47.5
60 years or older	53	38.1
**Education level**		
Illiterate	7	5.4
Middle school	59	45.4
High school	40	30.8
Higher education	24	18.5
**Alcohol consumption**		
No	57	43.8
Yes	73	56.2
**New case**		
No	28	20.1
Yes	111	79.9
**Clinical form**		
Cutaneous	113	81.3
Mucosal	15	10.8
Mucocutaneous	11	7.9
**Comorbidities**		
No	55	39.6
Yes	84	60.4
**Polypharmacy**		
No	125	89.9
Yes	14	10.1
**Potential drug-drug interactions**		
No	117	84.2
Yes	22	15.8
**Number of lesions**		
Up to 3	103	74.6
4 or more	20	14.5
Exclusively mucosal leishmaniasis	15	10.9
**Treatment**		
IV-MA	54	38.8
IL-MA	37	26.6
MILT	32	23.0
L-AMB	11	7.9
Others#	5	3.6
**AE occurrence**		
No	47	33.8
Yes	92	66.2
**Number of AEs**		
None	47	33.8
Up to 4	72	51.8
5 or more	20	14.4
**Severity of AE**		
Mild	249	78.3
Moderate	52	16.3
Severe	13	4.1
Life-threatening	3	0.9
Death	1	0.3
**Seriousness of AE**		
Nonserious	301	94.7
Serious	17	5.3
**Causality of AE**		
Certain	7	2.2
Probable/likely	101	31.8
Possible	195	61.3
Unlikely	12	3.8
Conditional/unclassified	2	0.6
Not assessable/unclassifiable	1	0.3
**Treatment discontinuation**		
No	129	92.8
Yes	10	7.2

**Abbreviations: AE:** adverse event. **IL-MA:** intralesional meglumine antimoniate; **IV-MA:** intravenous meglumine antimoniate; **L-AMB:** liposomal amphotericin B; **MILT:** miltefosine. ^#^ **Others:** meglumine antimoniate + pentoxifylline, fluconazole, and intralesional meglumine antimoniate + paromomycin.

**TABLE 2:** Profile of adverse events described by the High-Level Group Term and System Organ Classes, stratified by treatment used and seriousness (N = 318 events).

SOC or HLGT classification	Total number	L-AMB (n = 29)		IV-MA (n = 127)		IL-MA (n = 23)		MILT (n = 119)		Others^#^ (n = 20)	
		S	NS	S	NS	S	NS	S	NS	S	NS
**Gastrointestinal disorders**	**80**		**4**		**13**		**1**	**1**	**60**		**1**
Gastrointestinal ulceration and perforations		-	-	-	-	-	-	1	1	-	-
Other gastrointestinal disorders		-	4	-	13	-	1	-	59	-	1
**Investigations**	**75**	**1**	**6**	**6**	**33**		**1**	**1**	**17**	**1**	**7**
Gastrointestinal investigations		-	-	6	21	-	1	-	-	1	2
Hepatobiliary investigations		1	2	-	4	-	1	1	2	-	2
Other investigations		-	4	-	8	-	1	-	15	-	3
**Musculoskeletal and connective tissue disorders**	**43**		**1**		**27**		**2**		**9**		**4**
**Nervous system disorders**	**34**		**4**	**1**	**18**		**6**		**5**		
Seizures (including subtypes)		-	-	1	-	-	-	-	-	-	-
Other nervous system disorders		-	4	-	18	-	6	-	5	-	-
**General disorders and administration site conditions**	**23**		**3**		**5**	**1**	**7**		**3**		**4**
Fatal outcomes		-	-	-	-	1	-	-	-	-	-
Other general disorders		-	3	-	5	-	7	-	3	-	4
**Skin and subcutaneous tissue disorders**	**15**		**1**		**6**		**2**		**5**		**1**
**Cardiac disorders**	**8**		**1**	**1**	**4**		**1**	**1**			
Heart failures		-	-	1	-	-	-	-	-	-	-
Coronary artery disorders		-	-	-	-	-	-	1	-	-	-
Other cardiac disorders		-	1	-	4	-	1	-	-	-	-
**Infections and infestations**	**8**			**1**	**3**				**3**		
Bacterial infectious disorders		-	-	1	-	-	-	-	1	-	-
Other infections and infestations		-	-	-	3	-	-	-	2	-	1
**Respiratory, thoracic, and mediastinal disorders**	**6**		**2**		**3**				**1**		
**Blood and lymphatic system disorders**	**5**							**1**	**4**		
White blood cell disorders		-	-	-	-	-	-	1	-	-	-
Other blood disorders		-	-	-	-	-	-	-	4	-	-
**Immune system disorders**	**4**		**3**		**1**						
**Metabolism and nutrition disorders**	**7**	**1**			**2**				**3**		**1**
Electrolyte and fluid balance conditions		1	-	-	-	-	-	-	1	-	-
Other metabolism and nutrition disorders		-	-	-	2	-	-	-	2	-	1
**Renal and urinary disorders**	**4**		**1**		**1**				**2**		
**Eye disorders**	**3**				**2**				**1**		
**Ear and labyrinth disorders**	**1**								**1**		
**Psychiatric disorders**	**1**								**1**		
**Vascular disorders**	**1**		**1**								
**TOTAL (%)**	**318 (100%)**	**2**	**27**	**9**	**118**	**1**	**22**	**4**	**115**	**1**	**19**

**Abbreviations: HLGT:** High-Level Group Term; **IL-MA:** intralesional meglumine antimoniate; **IV-MA:** intravenous meglumine antimoniate; **L-AMB:** liposomal amphotericin B; **MILT:** miltefosine; **NS:** nonserious; **S:** serious; **SOC:** System Organ Classes.

^#^ **Others:** meglumine antimoniate + pentoxifylline, fluconazole, and intralesional meglumine antimoniate + paromomycin.

Although intravenous meglumine antimoniate (IV-MA) was the most used treatment (38.8%), miltefosine showed the highest rate of AEs in this population (3.7; Table 3). More AEs occurred among patients with the mucosal form of the disease (88.5%), and among the 113 patients with CL; more AEs occurred among those with four or more lesions (75.0%) than among those with up to three lesions (60.2%). All 10 patients whose treatment was discontinued due to a decision by the medical team reported at least one AE, of which 30.0% were classified as severe.

**TABLE 3:** Treatments used by the population, frequency of adverse events, serious adverse events, and the respective occurrence rates per patient.

Treatment	Number of patients (%)	Number of SAEs (%)	Number of reported AEs (%)	AE rate per patient	SAE rate per patient
IV-MA	54 (38.8)	9 (52.9)	127 (39.9)	2.3	0.16
IL-MA	37 (26.6)	1 (5.9)	23 (7.2)	0.6	0.03
MILT	32 (23.0)	4 (23.5)	119 (37.4)	3.7	0.12
L-AMB	11 (7.9)	2 (11.8)	29 (9.1)	2.6	0.18
Others^#^	5 (3.6)	1 (5.9)	20 (6.3)	*	*
**TOTAL**	**139 (100)**	**17 (100)**	**318 (100)**	**2.3 (100)**	**0.12 (100)**

**Abbreviations: AE:** adverse event; **IL-MA:** intralesional meglumine antimoniate; **IV-MA:** intravenous meglumine antimoniate; **L-AMB:** liposomal amphotericin B; **MILT:** miltefosine; **SAE:** serious adverse event. * Rate not calculated due to n < 10. ^#^ Others: meglumine antimoniate + pentoxifylline, fluconazole, and intralesional meglumine antimoniate + paromomycin.

The rate of serious adverse events (SAEs) was 0.12 (n = 17), with the highest rate observed among patients treated with liposomal amphotericin B (L-AMB; 0.18) and the lowest among those treated with intralesional meglumine antimoniate (IL-MA; 0.03; Table 3). The results of the analysis based on the High-Level Group Term classification (Table 2) showed that the most frequently occurring SAEs were related to gastrointestinal (n = 7) and hepatobiliary (n = 2) investigations. Most SAEs were considered related to the treatment received (70.6%). One fatal outcome (0.3%) was recorded among the 37 patients treated with IL-MA. However, this event was considered unrelated to treatment (unlikely) because multiple preexisting comorbidities were present and the lack of temporal plausibility.

In the analysis using logistic regression only, the treatment showed a significant association with AEs: a reduced likelihood of AEs was observed with IL-MA compared with IV-MA (odds ratio = 0.15; 95% confidence interval: 0.04-0.50; Supplementary Table S2).

The findings of this study indicate that a high incidence of AEs was associated with CL treatment, with mild gastrointestinal disorders being the most common, particularly among patients treated with miltefosine. Although IV-MA was the most prescribed regimen, the highest AE rate per treated patient was observed among those treated with miltefosine. AE occurrence varied according to the medication used, and IL-MA was associated with a lower likelihood of AEs than the intravenous route was.

The incidence of AEs identified in this study (66.2%) was substantially higher than that reported in another investigation evaluating a Brazilian population (6.6%)^9^. This difference may be explained, first, by the well-recognized toxicity of drugs used to treat CL^1^ and, second, the characteristics of the study setting, which promotes systematic AE recording and reporting, and thereby reduces the issue of underreporting^10^.

The safety profile of each treatment in this study was in line with those previously described, with most events classified as mild^11^. For IL-MA, local reactions at the site of administration predominated - an expected finding previously reported in a clinical trial^12^. For IV-MA, the events observed also agree with a profile described in the literature; that profile highlights investigations among the most frequent AEs, in addition to nervous system disorders such as headache^13^.

Regarding the AE profile of treatments with L-AMB, abnormalities in laboratory test results predominated. These findings differ from those of a previous review, in which infusion-related reactions were identified as the most frequent events^14^. This discrepancy may be explained by the characteristics of the service our center provides: L-AMB is not administered at our center, and patients are received only after the medication is administered. The use of miltefosine, in turn, showed the highest AE rate among the treatments, and these primarily involved gastrointestinal disorders. These events are already described in the literature^11^, and are therefore considered expected and manageable by recommending that the medication is administration with food.

Although not predominant in the study population, SAEs occurred more frequently with systemic treatments using IV-MA and L-AMB. Similar to the findings of another Brazilian study, the SAEs observed with IV-MA use were mainly related to gastrointestinal investigations findings; the other study reported that the drug was associated with a serious elevation in the lipase level^14^. Regarding SAEs associated with L-AMB use, the findings differ from those reported in the literature because we observed no renal toxicity^13^. This reflects the profile of our center, which only receives patients after treatment completion, a circumstance that possibly contributes to data loss between healthcare facilities. Of note, is that most of the SAEs reported were considered to be related to the treatment based on the WHO-UMC algorithm, thus supporting the known toxicity of the drugs used for CL^8^.

The occurrence of AEs in CL varies according to the treatment used, with local therapy using meglumine antimoniate showing the lowest number of events compared to therapy using IV-MA. This finding confirms the greater toxicity associated with systemic use of the drug, in line with a clinical study conducted in Brazil which reported a substantially higher number of AEs following systemic than local administration^15^.

This study had some limitations that should be considered when interpreting the results. First, the analyzed population was defined for convenience and included only individuals treated at the reference center. The study population may not represent the broader population of Brazil affected by CL because the reference center largely receives more complex cases that are referred from healthcare services across the region; therefore, no external validity is present. Second, due to the observational design of this study, causal inferences between the treatment used and AE occurrence could not be established. The findings should therefore be interpreted as associations observed during the study period, and they may have been influenced by individual patient characteristics acting as potential confounders. Third, the study was not designed to include inferential comparisons between subgroups.

Overall, the findings provide valuable evidence on pharmacovigilance in CL treatment, emphasizing the importance of tailoring therapeutic approaches to individual patient’s clinical characteristics to optimize treatment safety and efficacy. Continuous and structured monitoring of AEs is essential to improve patient safety, guide therapeutic choices, and strengthen post-marketing surveillance.

## Data Availability

Research data is only available upon request.
