# Supplementary material for: Pharmacovigilance in the treatment of cutaneous leishmaniasis: adverse event profile at a reference center
Source: Rev Soc Bras Med Trop. 2026 Sep 18;59:e0038-2026. doi: 10.1590/0037-8682-0038-2026 (PMC13592860; doi:10.1590/0037-8682-0038-2026)
Supplement: Supplementary file 2 [file 1678-9849-rsbmt-59-e0038-2026-md2.pdf]

Revista da Sociedade Brasileira de Medicina Tropical
Journal of the Brazilian Society of Tropical Medicine

SUPPLEMENTARY TABLE S2: Association between the occurrence of adverse events and the sociodemographic, lifestyle, clinical, and quality of life characteristics of the population.

Variables	Crude model OR (CI 95%)	p-value	Adjusted model OR (CI 95%)	p-value
**Sex**				
Female	1,00	0,632	1,00	0,394
Male	0,82 (0,36-1,85)		0,56 (0,15-2,12)	
**Age group (years)**				
15 to 29 years	1,00		1,00	
30 to 59 years	1,75 (0,63-4,87)	0,282	3,07 (0,59-15,84)	0,180
60 years or more	1,73 (0,60-4,97)	0,306	2,78 (0,32-23,88)	0,351
**Education level**				
Iliterate	1,00		1,00	
Middle school	0,30 (0,03-2,68)	0,282	0,34 (0,02-5,43)	0,444
High school	0,50 (0,05-4,67)	0,543	1,89 (0,10-35,54)	0,669
Higher education	0,33 (0,03-3,26)	0,345	0,62 (0,03-13,17)	0,763
**Alcohol consumption**				
No	1,00	0,920	1,00	0,686
Yes	1,04 (0,50-2,15)		1,26 (0,40-3,95)	
**New case**				
No	1,00	0,127	1,00	0,182
Yes	0,47 (0,17-1,24)		0,25 (0,03-1,90)	
**Clinical form**				
CL	1,00	**0,014**	1,00	0,242
ML	4,89 (1,38-17,25)		5,77 (0,31-108,42)	
**Comorbidities**				
No	1,00	0,607	1,00	0,513
Yes	1,21 (0,59-2,47)		1,54 (0,42-5,69)	
**Polypharmacy**				
No	1,00	0,184	1,00	0,499
Yes	0,47 (0,15-1,43)		0,51 (0,07-3,60)	
**Potential drug-drug interactions**				
No	1,00	0,237	1,00	0,971
Yes	1,90 (0,66-5,53)		1,05 (0,07-15,99)	
**Number of lesions**				
Up to 3	1,00	0,216	1,00	0,857
4 or more	1,98 (0,67-5,88)		0,86 (0,16-4,62)	
**Treatment**				
IV-MA	1,00		1,00	
IL-MA	0,15 (0,06-0,38)	**0,000**	0,15 (0,04-0,50)	**0,002**
MILT	5,25 (1,11-24,87)	**0,037**	5,02 (0,42-60,09)	0,203
L-AMB	0,42 (0,11-1,59)	0,202	0,16 (0,01-2,66)	0,199
**VAS**				
Below the population mean	1,00	**0,027**	1,00	0,131
Above the population mean	0,37(0,15-0,89)		0,36 (0,10-1,36)	

Legend: OR: Odds ratio and 95% confidence interval. Crude model: no adjustment for confounders. Adjusted model: adjustment for sociodemographic variables, lifestyle, clinical variables, and VAS. Legend: CL: cutaneous leishmaniasis. ML: mucosal leishmaniasis. IV-MA: intravenous meglumine antimoniate. IL-MA: intralesional meglumine antimoniate. MILT: miltefosine. L-AMB: liposomal amphotericin B. VAS: visual analog scale of quality of life.
